# Management and Surgical Repair of Neonatal Sternal Cleft: A Case Report of Two Successful Interventions

**DOI:** 10.7759/cureus.68327

**Published:** 2024-08-31

**Authors:** Muhammad Taimour Khan, Ibrahim Amjad, Muhammad Rahab Khan

**Affiliations:** 1 Internal Medicine, Institute of Dentistry, CMH Lahore Medical College, Lahore, PAK; 2 Plastic Surgery, AmjadPlastics, Miami, USA; 3 Internal Medicine, I.K. Akhunbaev Kyrgyz State Medical Academy, Bishkek, KGZ

**Keywords:** congenital malformation, genetic defect, cleft sternum, chest wall repair & reconstruction, sternal defect

## Abstract

The sternal cleft (SC) is a rare congenital anomaly characterized by a complete or partial separation of the sternum, leading to significant clinical concerns, including respiratory and cardiac instability. Due to its rarity, the SC often poses surgical challenges. This case report highlights the management of two neonates with SCs, emphasizing the critical role of early multidisciplinary intervention.

The first patient, a neonate, was born with a severe sternal deformity identified as a partial superior SC with a supraumbilical raphe. The patient was transported to a tertiary care center for further evaluation and management by a multidisciplinary team. Similarly, the second patient, another neonate, presented with similar sternal changes. Both patients were assessed for potential complications associated with sternal instability.

The treatment involved surgical correction by closing the gap of the SC for both patients. The procedure included a midline skin incision, dissection and lateral reflection of the pectoralis major muscle, resection of cartilaginous plates for healthy cartilage fusion, and extension of the cleft through the manubrium to achieve anatomic closure by approximation and suturing. Intraoperative monitoring ensured the stability of cardiac and respiratory functions. Postoperative outcomes were favorable, with both patients recovering well and being discharged without complications on postoperative days 5 and 9.

These cases highlight the significance of early surgical intervention with multidisciplinary management in neonates with SCs. The successful outcomes underscore the effectiveness of surgical intervention in preventing possible complications, ensuring rapid recovery, and stabilizing the chest. Further research into long-term outcomes and potential genetic factors may provide deeper insights into the management of this rare condition.

## Introduction

Congenital sternal cleft (SC) is a rare disorder of unknown origin characterized by an opening in the sternum that exposes mediastinal organs and vessels to injury [1]. It results from incomplete fusion of the mesodermal lateral plates due to a failure in midline development [2]. This incomplete fusion can occur between the sixth and seventh weeks of gestation, leading to improper fusion of the mesenchymal plates [2-3]. A partial or total failure of this fusion during early embryological development results in a congenital SC [4].

SCs can be classified as either complete or partial. A complete SC involves a split along the entire length of the sternum, creating a continuous gap from top to bottom [3]. A partial SC affects only a portion of the sternum and can be further subdivided into superior partial SC and inferior partial SC [1,5]. Each form can occur as an isolated defect or in association with other congenital deformities, such as vascular dysplasia, PHACES syndrome, midline fusion defects, and Cantrell’s pentalogy [1]. These associations present unique challenges for reconstructive surgeons.

PHACES syndrome is a congenital anomaly in which hemangiomas present with systemic anomalies, as described by the acronym PHACE coined by Frieden et al. in 1996. The acronym PHACE included Posterior fossa brain abnormalities, Hemangiomas that may involve the face, Arterial cerebrovascular lesions, Cardiovascular anomalies such as aortic coarctation, and Eye abnormalities but later it was changed to PHACES to include SC or supraumbilical raphe, which are ventral developmental defects [1,6]. PHACES syndrome is noteworthy among congenital anomalies associated with SC, as it is more common than recognized by clinicians, with 20% of cervicofacial hemangiomas being linked with PHACES syndrome [6].

Typically, a skin defect overlying the SC is covered by a white translucent membrane, through which the beating of the heart and protrusion of soft tissue can be seen during expiration. The right heart chamber, covered by the pericardium, is in contact with this skin defect [3]. Paradoxical chest wall movement is observed, with a bulge seen during expiration and depression during inspiration, as well as during crying or coughing [3].

SC is a rare congenital anomaly, accounting for only 0.15% of all chest wall abnormalities, with an estimated incidence of 1 in 50,000 to 100,000 live births [3,7]. Superior partial SC is the most common form of partial SC, while inferior partial SC is even less common [5].

Diagnosis is typically made using CT imaging, which also helps assess the extent of the defect [3]. Treatment involves closing the gap in the sternum. Various surgical interventions can be employed, with the most common method being primary closure [1]. Other methods include bone graft interposition, prosthetic closure, and muscle flap interposition [1]. Primary closure is particularly recommended during the neonatal period due to the compliant thorax, which facilitates maneuverability and suturing [8]. Nonetheless, primary closure should be considered regardless of age, with alternative surgical methods depending on the size of the SC, the suitability of the method, and the stability of the patient [5].

In this report, we present a case involving two infants, aged 22 days and nine days, who underwent surgical operations for partial SCs. We focus on pre-operative evaluations, challenges posed by complicating factors, and the approach taken for surgical management. These cases highlight the importance of early recognition and intervention in improving patient outcomes.

## Case presentation

Case 1

**Figure 1:**
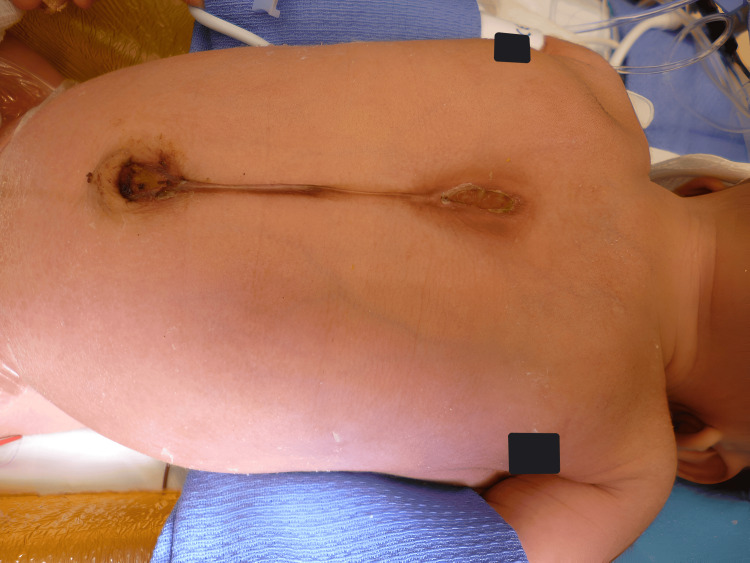
Supraumbilical raphe, a midline defect in the chest, and laterally displaced nipples (obscured in black boxes).

The patient presented in an outlying facility due to concerns about changes in the sternum and potential sternal instability. A full evaluation was done by specialists in plastic surgery, cardiothoracic surgery, neonatology, and cardiology. A supraumbilical raphe was noted with a sternal defect as seen in Figure 1, but no underlying deformities or syndromic changes were observed. The patient was monitored in the neonatal intensive care unit. CT scan showed a sternal defect extending to the fourth rib. Respiratory difficulty was noticed with paradoxical movement and pronounced respiratory effort. Considering the urgency, the patient was scheduled for surgical repair on the 10th day of life.

**Figure 2:**
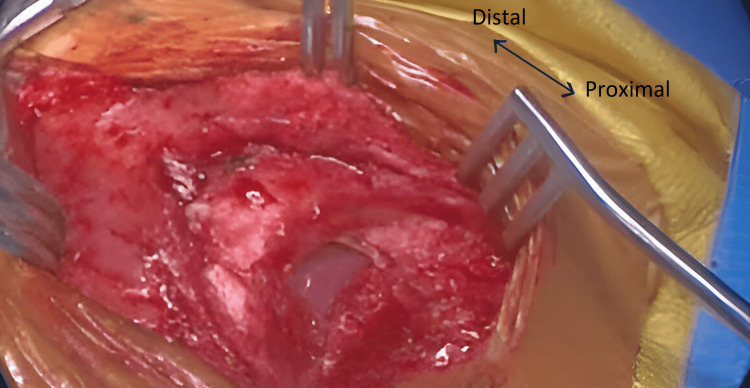
Midline incision and view of cleft sternum encountered through lateral retraction with an arrow highlighting the orientation of the cleft from proximal to distal.

**Figure 3:**
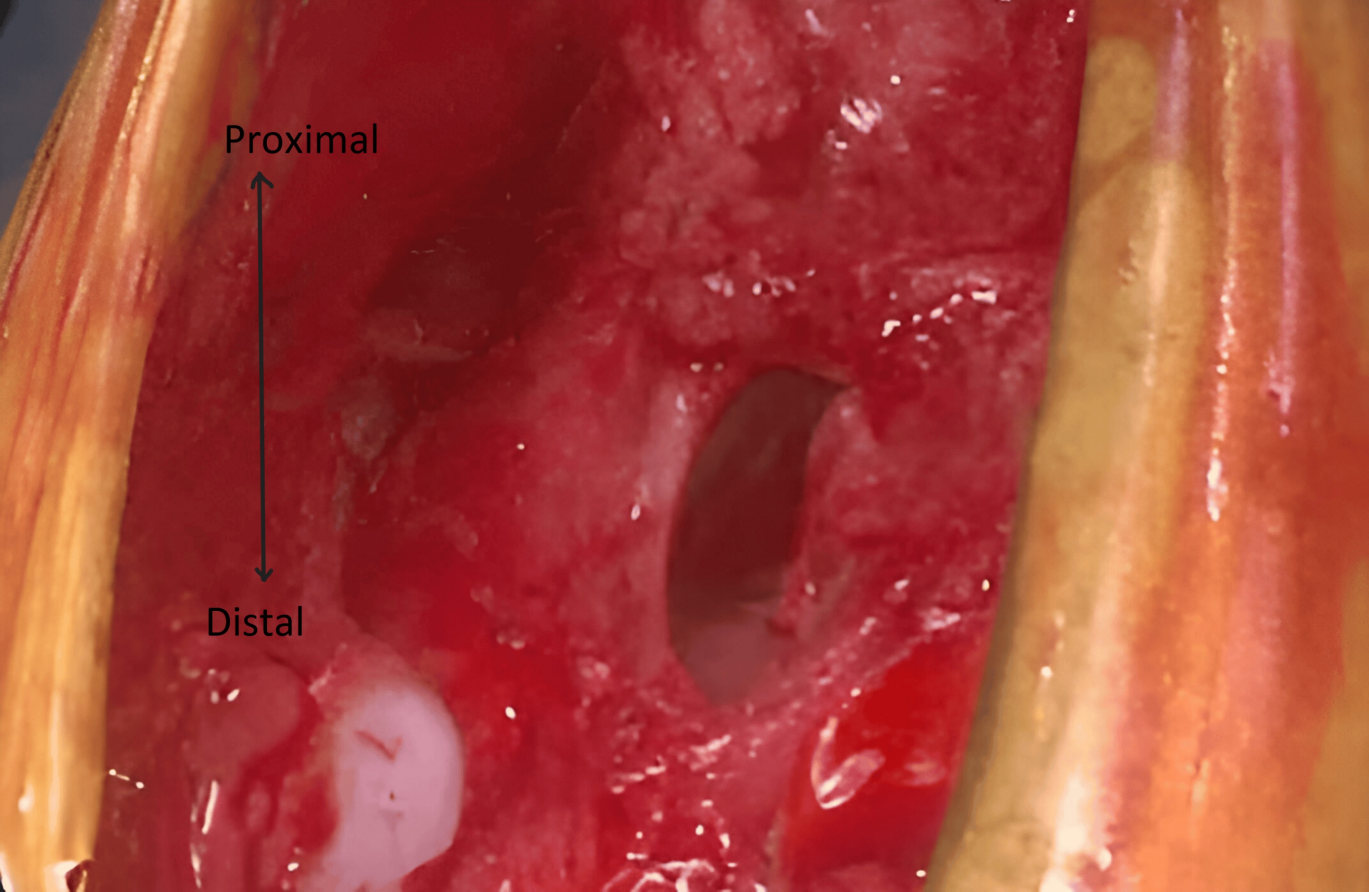
SC with a defect in the pericardium membrane from proximal to distal end (as highlighted by arrow). SC, sternal cleft

The procedure was performed with the patient in the supine position. Through a midline approach, the skin was opened. The pectoralis major muscle was dissected and reflected laterally. During this process, a partial SC was encountered long with a central midline defect as seen in Figure 2 and Figure 3. 

**Figure 4:**
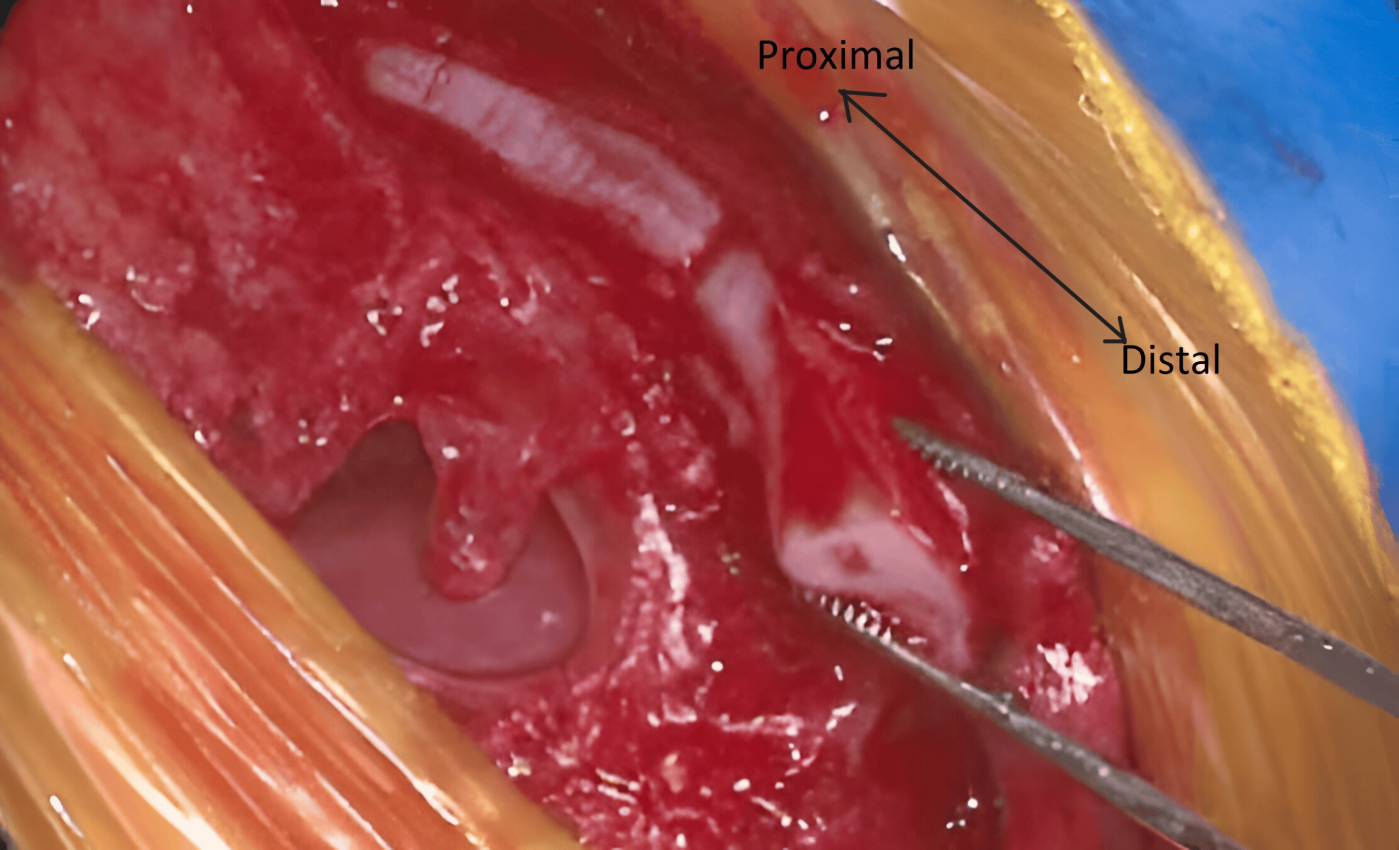
A cartilaginous plate held by forceps at the distal end with an arrow highlighting the orientation from proximal to distal end. This is after the extension of the cleft and complete separation of the medial ends of the cleft sternum.

Through the incision, the defect was extended vertically through the point of fusion of the sternum below achieving complete midline separation of the sternal ends. To achieve repair, resection of 2 mm of the cartilaginous plate on both medial sides of the defect was performed to attain healthy cartilage to facilitate fusion. The separated and shaved medial cartilage of one side of the sternum can be seen in Figure 4. 

**Figure 5:**
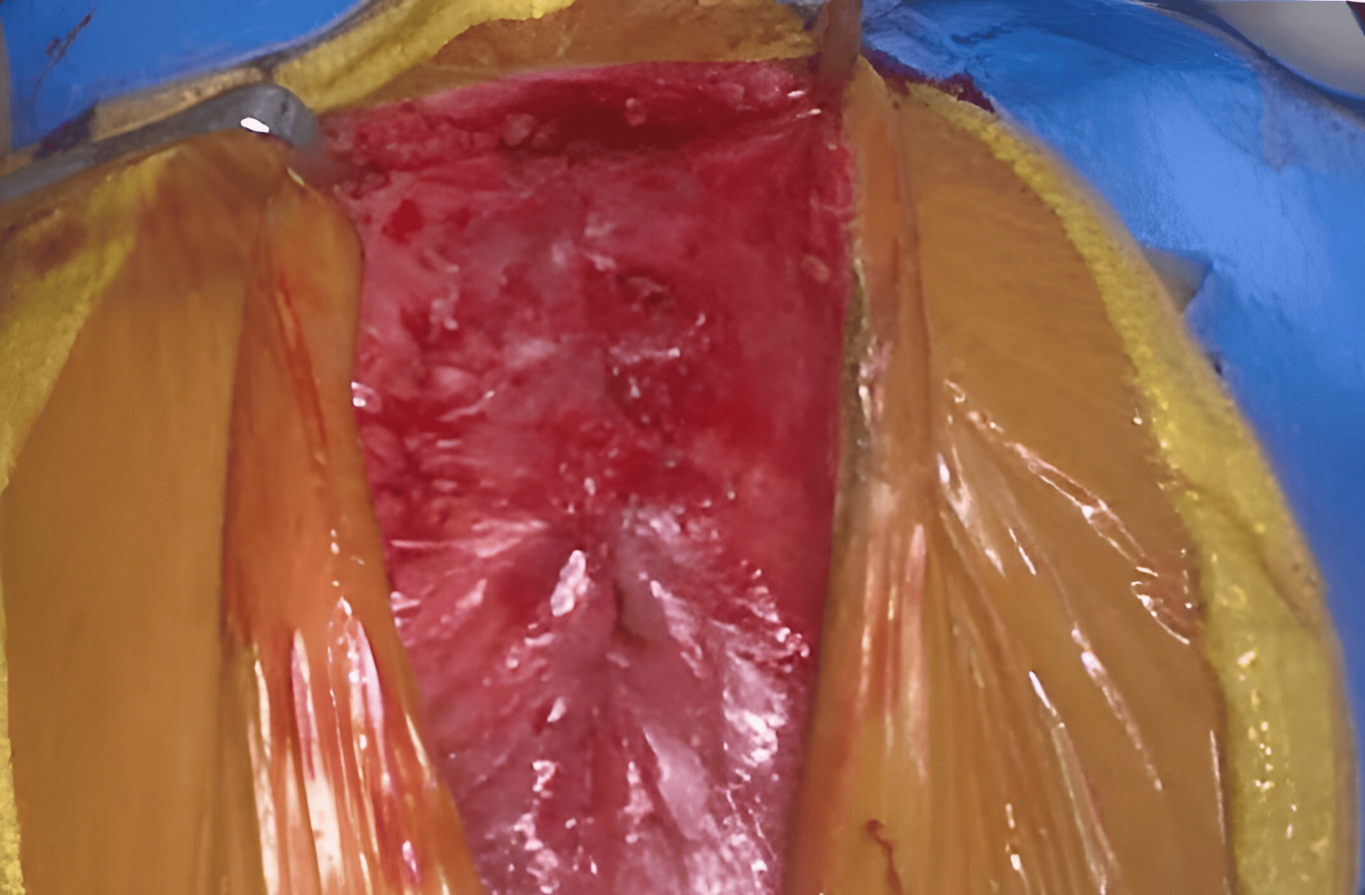
Primary closure of SC by approximation and suture of cartilaginous plates. SC, sternal cleft

**Figure 6:**
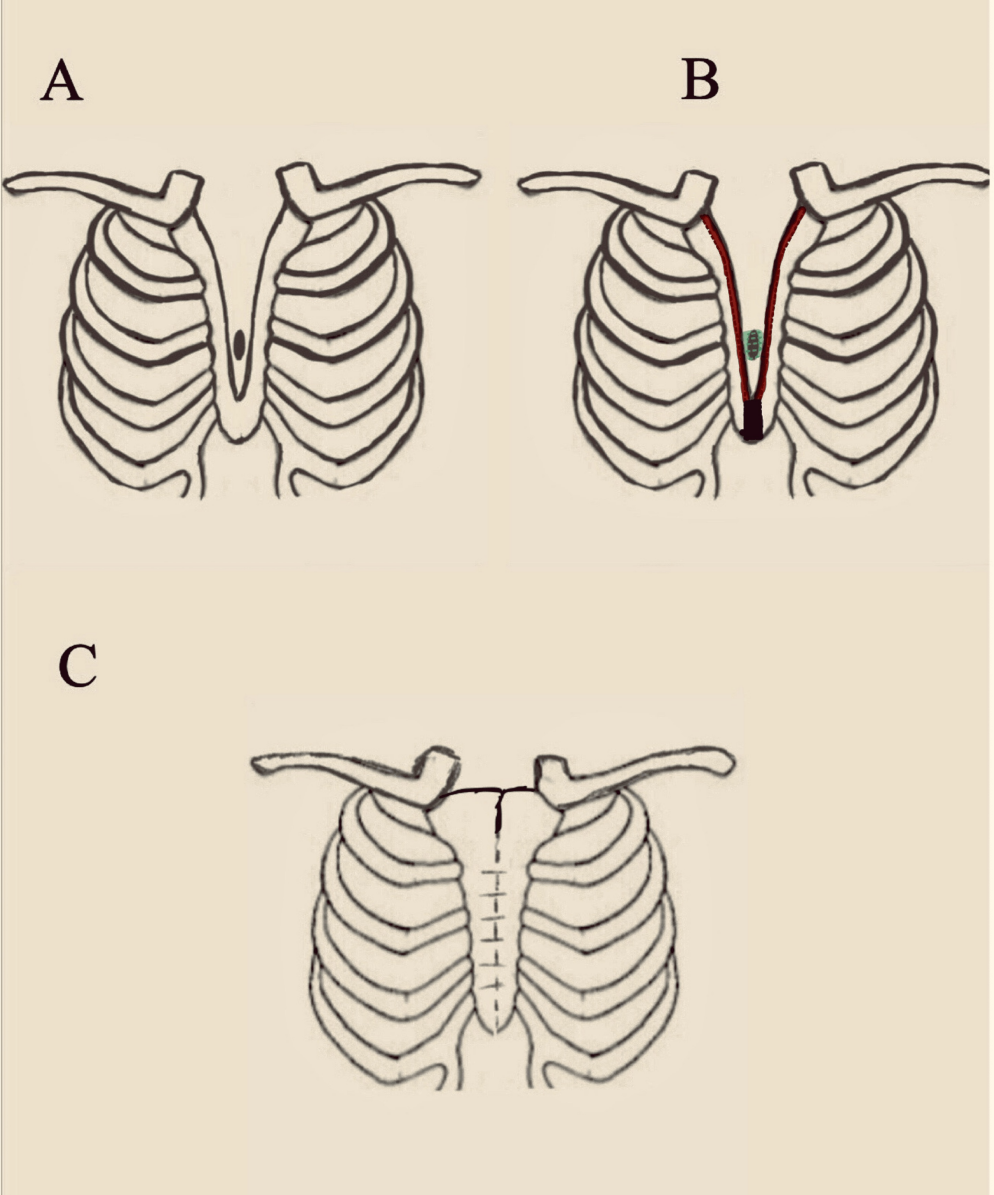
Procedure used for cleft sternum repair. A illustrates the defect encountered. B shows the process, where the area shaded in green is sutured and closed, the edges of the sternum shaded in red are shaved to expose fresh cartilage, and the area shaded in black is removed. C demonstrates the alignment of the fresh ends of the separated plates, brought together through suturing.

After juxtaposing the margins through mechanical support, the gap was closed. The pectoralis muscle was mobilized to the midline to provide full coverage of the repair, achieving primary closure as seen in Figure 5. The steps involved in the process of repair are illustrated in Figure 6. 

Case 2

**Figure 7:**
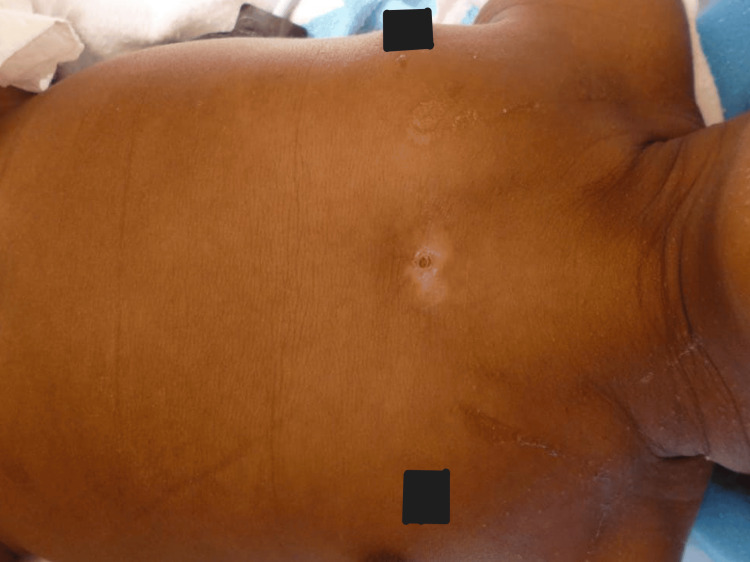
Presentation of SC with a skin defect and laterally displaced nipples (obscured in black boxes). SC, sternal cleft

The patient presented in a similar fashion in an outlying hospital and was noted to have a severe deformity of the sternum as can be seen in Figure 7. The patient was emergently transported due to this malformation and concerns about possible complications. The patient was assessed by plastic surgery, cardiology, cardiothoracic surgery, and neonatology specialists. No other syndromic changes were observed. Imaging studies revealed a partial superior SC extending to the fourth rib. There was concern regarding sternal instability and potential respiratory issues. The patient was monitored for 10 days. Considering the benefits of early surgical correction, the patient was scheduled for surgery at 22 days of age after a comprehensive discussion in a multidisciplinary conference.

**Figure 8:**
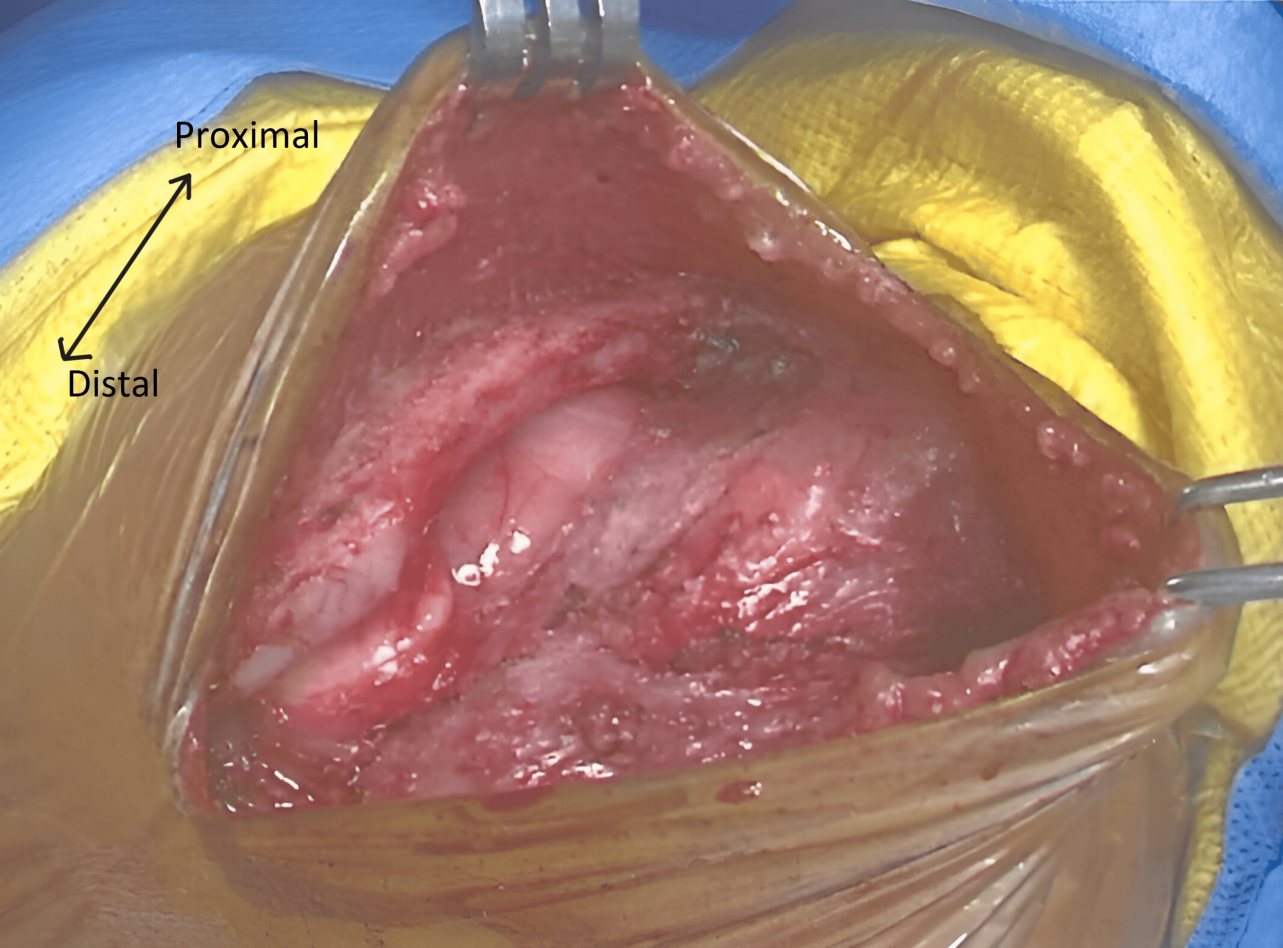
View of the SC encountered, visualized through a midline incision with lateral skin retraction with an arrow highlighting proximal to distal orientation. SC, sternal cleft

Similar to the technique used for patient 1, primary closure was the technique of choice. In a supine position, a midline incision was made and extended with the dissection of the pectoralis major. A SC was encountered with the widening of soft tissue by retractors as seen in Figure 8.

**Figure 9:**
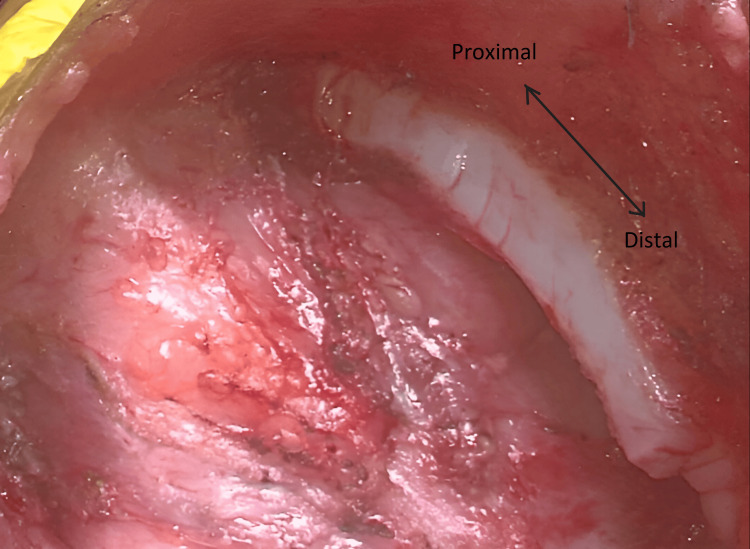
Shaved medial end of the sternal cartilage with excised lower end to achieve complete separation with an arrow highlighting proximal to distal orientation.

**Figure 10:**
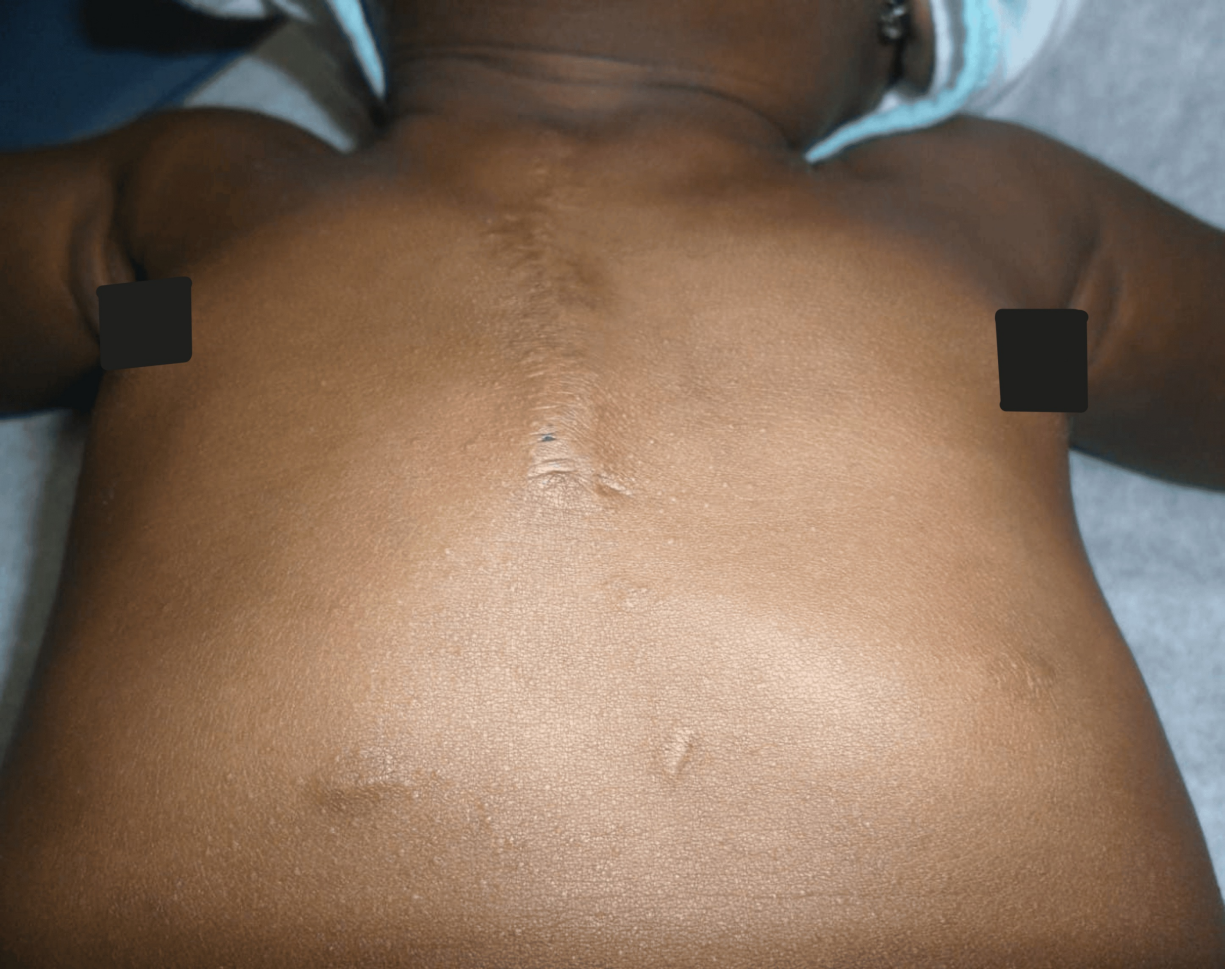
Postoperative view of the chest.

Similar to the process illustrated in Figure 6, the cleft in the sternum was extended vertically resulting in complete separation of the sternum. The medial edges of the sternum were shaved for fresh cartilage as seen in Figure 9 and the ends were aligned and reinforced through suturing. The patient was kept for observation and discharged nine days after the operation. The postoperative image of the patient can be seen in Figure 10.

## Discussion

Both patients presented with partial SCs and concerns about potential sternal instability, which led to early surgical intervention to close the gap. The primary closure method was used, involving bringing the edges of the cleft together and securing them with sutures and other materials. This method is often preferred when the gap is small and the tissues are flexible enough to be joined without excessive tension [5,8,9]. For proper fusion, the cartilaginous tissue on either side was trimmed to ensure healthy fusion. By extending the closure through the manubrium and mobilizing the pectoralis major muscles to the midline, complete anatomical closure and successful primary closure of the partial SC in both cases were achieved.

Primary closure is considered the easiest technique and is preferable for treating SCs in young patients [9]. Early surgery for the SC is recommended, especially within the first four weeks of life [10]. A common complication resulting from this procedure is the development of a slight degree of pectus excavatum, which typically occurs long-term and usually does not require surgical intervention [9].

Patients undergoing primary closure may also have a partial thymectomy to provide better access during the procedure. This helps reduce interference from the thymus, improving visualization and manipulation of the tissue around the sternum and reducing complications [11]. Other methods may involve reinforcing the primary closure with titanium mesh to provide better stability and reduce tension at the closure site; titanium mesh is preferred due to its osseointegration properties [10]. The use of Marlex mesh is also noted for its advantages in reducing cardiorespiratory issues and preventing septic or aseptic necrosis [12]. While primary closure is preferred during the neonatal period, using prosthetics can also yield good results [1]. However, primary closure is recommended regardless of age, and the choice of surgical method for SC can depend on the size of the gap [5].

## Conclusions

The presented cases of the two neonates with partial SCs demonstrate the effectiveness of early surgical intervention using primary closure techniques. The decision to perform early surgery, within the first four weeks of life, was crucial in managing the potential complications associated with sternal instability. The surgical approach involved trimming the cartilaginous tissue and mobilizing the pectoralis major muscles to achieve complete anatomical closure. This method is especially favorable in young patients due to the small gap size and the flexibility of the tissues, which allows for a secure and tension-free closure.

Although primary closure is generally the preferred method, especially in neonates, other techniques, such as the use of prosthetics or reinforcing materials like titanium mesh, may be considered based on individual patient characteristics and the size of the cleft. Overall, early diagnosis and prompt surgical intervention are key to achieving favorable outcomes in patients with SCs. Further research into long-term outcomes and alternative surgical techniques may provide additional insights into optimizing care for this rare congenital condition.
